# Structural Characterization and Anticoagulant Activity of a 3-*O*-Methylated Heteroglycan From Fruiting Bodies of *Pleurotus placentodes*

**DOI:** 10.3389/fchem.2022.825127

**Published:** 2022-01-27

**Authors:** Zhen-Hua Yin, Xiao-Peng Liu, Jin-Mei Wang, Xue-Feng Xi, Yan Zhang, Rui-Lin Zhao, Wen-Yi Kang

**Affiliations:** ^1^ National R & D Center for Edible Fungus Processing Technology, Henan University, Kaifeng, China; ^2^ College of Physical Education, Henan University, Kaifeng, China; ^3^ Hebei Food Inspection and Research Institute, Shijiazhuang, China; ^4^ State Key Laboratory of Mycology, Institute of Microbiology, Chinese Academy of Sciences, Beijing, China

**Keywords:** *Pleurotus placentodes*, polysaccharide, 3-*O*-methylated heteroglycan, structure identification, anticoagulant activity

## Abstract

*Pleurotus placentodes*, a fungus, belongs to the Pleurotaceae family. The aim of the present study was to characterize the structure of a novel polysaccharide from fruiting bodies of *P. placentodes* (PPp-W) and evaluate its anticoagulant activity *in vitro*. The high-performance liquid chromatography and GC–MS analysis indicated that PPp-W with a molecular weight of 27.4 kDa was mainly composed of mannose (17.56%), glucose (6.37%), galactose (44.89%), and fucose (1.22%) with a certain amount of 3-*O*-methyled galactose. SEM, XRD, and AFM combined with Congo red test revealed that PPp-W was an irregular curly sheet with triple-helix conformation. The FT-IR, methylation, and nuclear magnetic resonance analysis indicated that PPp-W contained→6)-α-D-Gal*p*-(1→, →6)-3-*O*-Me-α-D-Gal*p*-(1→and →2, 6)-α-D-Gal*p*-(1→ as main chain, partially substituted at *O*-2 and *O*-6 by non-reducing ends of β*-*D*-*Man*p*-(1→ and β*-*L-Fuc*p*-(1→ with a small amount of α-1,3-linked-Glc*p* in backbone. PPp-W could significantly prolong APTT (12.9 ± 0.42 s, *p* < 0.001) and thrombin time (39.9 ± 0.28 s, *p* < 0.01) compared with the control group (11.45 ± 0.071 s and 38.05 ± 0.21 s), which showed that PPp-W had anticoagulant activity. These studies suggested that PPp-W was a 3-*O*-methylated heteroglycan and might be suitable for functional foods and natural drugs as an anticoagulant ingredient, which provided a basis for the application of polysaccharides from *P. placentodes*.

## Introduction

In recent years, thrombotic diseases show an increasing trend every year; it is bad for the health of people. According to the World Health Organization, nearly 3.6 million people are expected to die from thrombotic diseases by 2030 (WHO, 2004). More and more people are affected by thrombotic diseases, which urges people to look for new anticoagulant products with fewer side effects. Heparin, the most commonly used anticoagulant, was a sulfate polysaccharide composed of uronic acid and glucosamine alternating chains, but it had some shortcomings such as hemorrhage and thrombocytopenia (Hirsh and Raschke, 2004). Polysaccharide, one of the natural active ingredients, also showed anticoagulant activity (Cai et al., 2016; Gunasekaran et al., 2021).

Several studies had demonstrated that *Pleurotus* spp. contained terpenoids, steroids, phenolic acid derivatives, polyyne, and especially polysaccharides, one of the active components, had antioxidant, antiaging, anti-inflammatory, immunomodulatory, antitumor, antimicrobial, and anti-obesity hypolipidemic and hypoglycemic activities (Barbasa et al., 2020; Sharma et al., 2021). *Pleurotus placentodes*, belonging to the genus *Pleurotus* spp., was first described by British mycologist Berkeley in 1852 (Berkeley, 1852), and had not been reported for more than 100 years. Until 2016, researchers from Kunming Institute of Botany of the Chinese Academy of Sciences found *P. placentodes* in subalpine forests at an altitude of 3,000–4,200 m in Xizang and Yunnan (China). This was the first report on *P. placentodes* in China (Liu et al., 2016). *P. placentodes* had a high content of proteins (30.1/100 g), crude polysaccharides (7.45/100 g), and amino acids (19.24/100 g) than of common commercially cultivated mushrooms such as *Lentinula edodes*, *P. ostreatus*, *Flammulina velutipes*, and *Cyclocybe aegerita* (Zhang et al., 2017; Wang et al., 2018). It was well known that the biological activities of polysaccharides were closely related to their molecular weight, monosaccharide composition, configuration, conformation, glycosidic linkage, and branching degree (Gong et al., 2020). However, there were no reports on the structure and biological activity of polysaccharides from *P. placentodes*.

In the current research, a polysaccharide was isolated from fruiting bodies of *P. placentodes*. The fine structure was identified by gas chromatography–mass spectrometer (GC–MS), high-performance liquid chromatography (HPLC), Fourier-transform infrared spectroscopy (FT-IR), and nuclear magnetic resonance (NMR), and the microstructure was detected by scanning electron microscope (SEM), X-ray diffractometer (XRD), atomic force microscope (AFM), and Congo red test. The anticoagulant activity was investigated by activated partial thromboplastin time (APTT), prothrombin time (PT), and thrombin time (TT) *in vitro*. These efforts were made to find polysaccharides with anticoagulant activity and promote the application of polysaccharides.

## Materials and Methods

### Materials and Reagents

Dried fruiting bodies of *P. placentodes* (voucher specimen number: 2020807) were provided by Institute of Microbiology, Chinese Academy of Sciences (Beijing, China) in August 2020, and were identified by Prof. Ruilin Zhao (Wang et al., 2018). Monosaccharides: D-mannnose (Man), L-rhamnose (Rha), D-glucose (Glc), D-xylose (Xyl), D-galactose (Gal), and D-arabinose (*Ara*) were purchased from Dr. Ehrenstorfern GmbH. D-Fucose (Fuc) and D-galacturonic acid (GalA) were purchased from Chrome dex, and D-glucuronic acid (GlcA) was purchased from Alfa Aesar. D-lyxose (Lyx) and 3-methyl-1-phenyl-2-pyrazoline-5-one (PMP) were purchased from Aldrich chemistry. Methyl iodide (CH_3_I) was purchased from Adamas Reagent, Ltd (Shanghai, China). Deuterium oxide (D_2_O) was provided by Sigma-Aldrich Chemical Co. Activated partial thromboplastin time (APTT) assay kit (20210803M), prothrombin time (PT) assay kit (20210601M), and thrombin time (TT) assay kit (20200113M) were purchased from Shenzhen Leidu Life Science Co., Ltd (Guangdong, China). Other reagents were analytically pure.

### Extraction and Purification of Polysaccharides From *P. placentodes*

The polysaccharides from fruiting bodies of *P. placentodes* were extracted and purified according to our previous method (Liang et al., 2021) with some modifications. First, the fruiting bodies were extracted with 70% ethanol, and then the residues were slightly boiled twice with water for 4 h. The extracting solution was concentrated to a density of 1.1 in vacuum at 55°C. The concentrate was slowly precipitated by adding 95% ethanol to reach a final concentration of 70%. After centrifugation (4,500 rpm, 6 min), the precipitate was collected and re-dissolved in ultrapure water, deproteinized by Sevage method, and lyophilized to give crude polysaccharides from *P. placentodes*.

**FIGURE 1:**
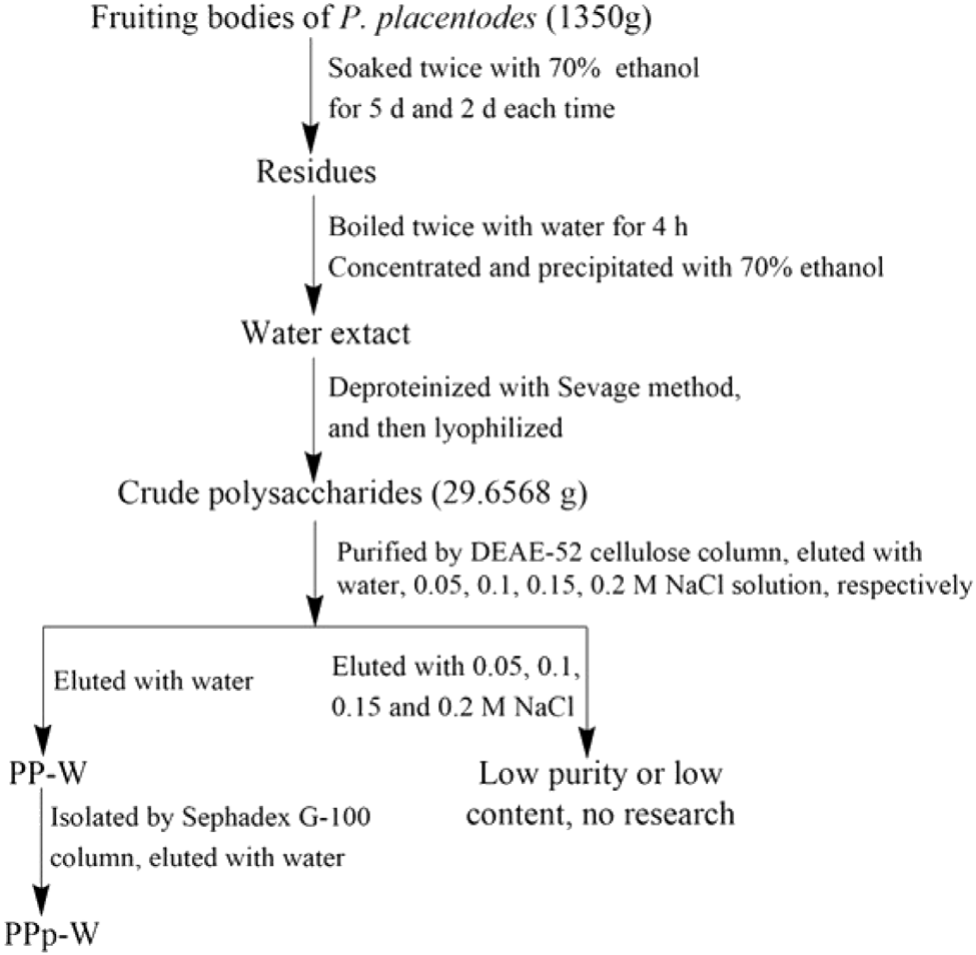
The flow chart of extraction and separation of polysaccharides from *P. placentodes*.

The crude polysaccharides were purified by DEAE-52 cellulose column (60 × 2.5 cm) and eluted with water and 0–0.2 M NaCl. The eluents were collected with a program-controlled multi-function automatic partial collector (Shanghai Huxi Analysis Instrument Factory Co. Ltd.). A tube was collected per 6 min; the eluents were colored with concentrated sulfuric acid–phenol. Due to the low contents of polysaccharides, NaCl eluents were not studied. Water eluents were combined to get the purified polysaccharides (PP-W). PP-W was further purified by Sephadex G-100 column (100 × 1.50 cm). According to the similar operation of DEAE-52 column chromatography, the eluents were collected, colored, and merged to get pure polysaccharides (PPp-W). The flow chart of extraction and separation was shown in Figure 1.

### Homogeneity and Molecular Weight

The homogeneity and average molecular weight (Mw) of PPp-W were determined by Shimadzu LC-20A HPLC system (Shimadzu Co., Ltd.), equipped with a 20A refractive index detector (RID) and a Shodex OHpak SB-806 HQ column (10 μm, 8 mm × 300 mm). The mobile phase was ultrapure water, flow rate was 0.5 ml/min, column temperature was 35°C, and injection volume was 40 μl. T-series dextran standards (Aladdin Biochemical Technology Co., Ltd., Shanghai, China) with different Mws (500.0, 200.0, 100.0, 40.0, 10.0, 5.0, and 1.0 KDa) and Glu (180 Da) were used to establish a calibration curve.

### Monosaccharide Compositions

The monosaccharide compositions and their mass ratios of PPp-W were determined by PMP–HPLC based on a previous report (Ma et al., 2020) with some modifications. PPp-W (6 mg) was hydrolyzed with 4 mol/L trifluoroacetic acid (TFA) at 110°C for 4 h. The hydrolysates were mixed with 240 μl water, 600 μl Lyx (0.1 mg/ml), 600 μl PMP methanol solution (0.5 M), and 240 μl NaOH (0.23 M); reacted at 70°C for 100 min; and neutralized with 400 μl HCl (0.15 M). The mixture was extracted with CH_3_Cl and filtered through 0.22 μm microporous membrane for HPLC. The standard monosaccharides were derivatized in the same way. The monosaccharide compositions were determined by Shimadzu LC-20A HPLC system (Shimadzu co. Ltd.), fitted with a SPD-20A UV/Vis detector and a TC-C18 column (250 mm × 4.6 mm, 5 μm). Mobile phase A and B were acetonitrile and sodium phosphate buffer (pH = 6.8), respectively, and flow rate was 1.0 ml/min, column temperature was 35°C, and injection volume was 10 μl. The monosaccharide content was calculated by internal standard method, and Lyx was used as the internal standard.

### Scanning Electron Microscopy (SEM) Analysis

The PPp-W powder was attached to the sample holder with copper tape, and was then plated with a layer of conductive gold powder in the ion sputtering instrument. The micromorphology was observed by SEM (FEI Quanta 250 FEG, Hillsboro, OR, United States). The acceleration voltage was 20 kV, and the observation multiples were 2,000×, 5,000×, and 10,000×, respectively.

### X-Ray Diffraction (XRD) Spectrum Analysis

The powder XRD patterns of PPp-W was investigated by X-ray diffractometer (D8 Advance, Bruker, Germany). The setting conditions are as follows: Cu target, Ka ray, scanning step 0.04°, scanning range 2θ = 5–80°, pipe flow 40 kV, RS, tube pressure 40 mA, RS 0.3 mm. Data were evaluated by software Jade 6.0.

### Congo Red Method

The triple helical structure of PPp-W was determined by the Congo red method according to (Zeng and Zhu, 2018) with some modifications. PPp-W (1 ml, 2 mg/L) was mixed with 1.5 ml of Congo red reagent (0.2 mM) and then bonded at different NaOH concentrations (0–0.8 M). The maximum absorption wavelength was measured in the range of 300–600 nm by ultraviolet–visible spectrophotometer.

### Atomic Force Microscopy (AFM) Analysis

PPp-W was dissolved and diluted into a solution with a concentration of 10 μg/ml. About 10 μl solution was dripped on the surface of mica sheet and dried naturally at room temperature. The AFM image was performed in tap mode using a NT-MDT Solver P47H-PRO atomic force microscope (Russian Federation).

### Fourier-Transform Infrared Spectroscopy (FT-IR)

PPp-W was ground with spectroscopic grade KBr powder, and then pressed into pellets for FT-IR spectrum analysis. The FT-IR spectrum was measured within a frequency range from 4,000 to 400 cm^−1^ on an iS50 FT-IR spectrometer (Thermo).

### Glycosyl Linkage Analysis

Methylation combined with GC–MS analysis was carried out to measure the glycosyl linkage of PPp-W, according to the previously reported method (Wang et al., 2019). In brief, PPp-W (5 mg) was dissolved in dimethyl sulfoxide (DMSO), followed by the addition of NaOH powder (25 mg) and CH_3_I (1 ml). Complete methylation was confirmed by the disappearance of the OH band (about 3,500 cm^−1^) in the FT-IR spectrum (Supplementary Figure S1). Then, the methylated products were hydrolyzed (4 mol/L TFA) and subjected to reduction (sodium borodeuteride) and acetylation (acetic anhydride) to derive partially methylated alditol acetates (PMAAs). PMAAs were analyzed by GC−MS using an Agilent Technology 8890/7000D TQ system (Agilent Technologies Corp., United States), equipped with a HP-5ms column (30 m × 0.25 mm, 0.25 μm film thickness). The initial temperature was 120°C; then, it was increased to 180°C at 5°C/min, held for 5 min, subsequently increased to 240°C at 5°C/min, and held for 3 min. PMAAs were identified by database and comparison with standards (Sassaki et al., 2005).

### NMR Spectroscopy

PPp-W (25 mg) was dissolved in 0.5 ml D_2_O, and then loaded into a 5-mm NMR tube. The ^1^H-NMR spectrum, ^13^C-NMR spectrum, ^1^H-^1^H correlation spectroscopy (COSY), ^1^H-^13^C heteronuclear single-quantum coherence spectroscopy (HSQC), and ^1^H-^13^C heteronuclear multiple-bond spectroscopy (HMBC) were recorded at 400 and 100 MHz, respectively, on a Bruker Avanced III 400 MHz NMR spectrometer (Brucker, Switzerland) with a 5 mm probe at 298 K.

### Anticoagulant Activity and Statistical Analysis

The study was conducted in accordance with the requirements of the Animal Ethics Committee of Henan University (HUSOM2021-076). Blood was collected from the abdominal artery of SD rats, and immediately mixed with 0.109 mol/L sodium citrate and centrifuged at room temperature for 12 min at 3,000 rpm; the upper plasma was used to determine the anticoagulant activity.

PPp-W was dissolved in normal saline to form a solution with a concentration of 3 mg/ml. Breviscapine (Bre) with an anticoagulant activity was configured to a concentration of 13.33 mg/ml. Normal saline was blank control (BC). Referring to our previous experimental method (Liang et al., 2021), according to the operation instructions of APTT, PT, and TT assay kits, the APTT, TT, and PT values were determined on a RAC-030 automatic coagulation analyzer (Shenzhen Leidu Life Science Co., Ltd. Guangdong, China).

All values were expressed as mean values ± standard deviation. Statistical analyses were carried out using SPSS 19.0 software. Difference between groups was analyzed by one-way analysis of variance (*p* < 0.05).

## Results and Discussion

### Purification, Molecular Weight, Monosaccharide Composition, and Homogeneity

**FIGURE 2:**
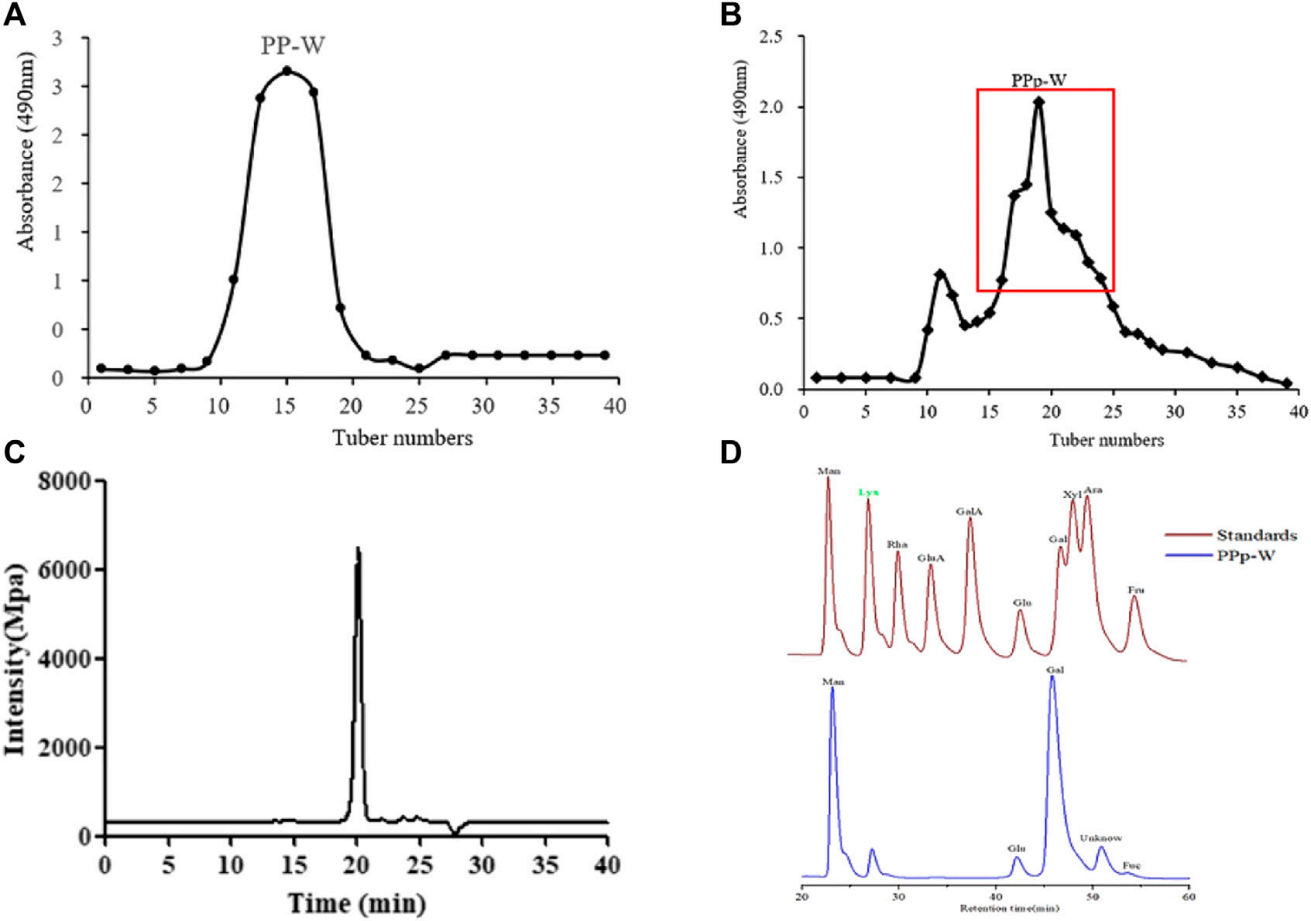
Elution curve of DEAE-52 column chromatography **(A)**. Elution curve of Sephadex G-100 column chromatography **(B)**. Homogeneity of PPp-W **(C)**. Monosaccharide composition of PPp-W **(D)**.

The crude polysaccharides were extracted from the fruiting bodies of *P. placentodes* by hot water extraction and alcohol precipitation followed by deproteinization. The yield of crude polysaccharides was 2.20%. A polysaccharide (PPp-W) was obtained by preliminary purification with DEAE-52 cellulose column chromatography (Figure 2A), and further purified by Sephadex G-100 column chromatography (Figure 2B). PPp-W was a white flocculent powder after freeze-drying, and was considered a neutral polysaccharide because it was eluted with water and did not contain acidic sugars (This had been proved in FT-IR analysis) (Hokputas et al., 2004). HPGPC results suggested a symmetrical peak was exited in PPp-W; the purity was more than 95% (Figure 2C), which indicated PPp-W had high homogeneity. The Mw was calculated to be about 27.4 kDa according to the established standard calibration curve (log^M*w*^ = −1.4874Rt + 34.399, *r* = 0.996 4). PMP–HPLC results (Figure 2D) illustrated that PPp-W contained Man, Glu, Gal, and Fuc in the percentages of 17.56, 6.37, 44.89, and 1.22% (mass ratio), which implied that PPp-W was a heteropolysaccharide.

### Morphological Characterization of PPp-W

#### Scanning Electron Microscopy Analysis

**FIGURE 3:**
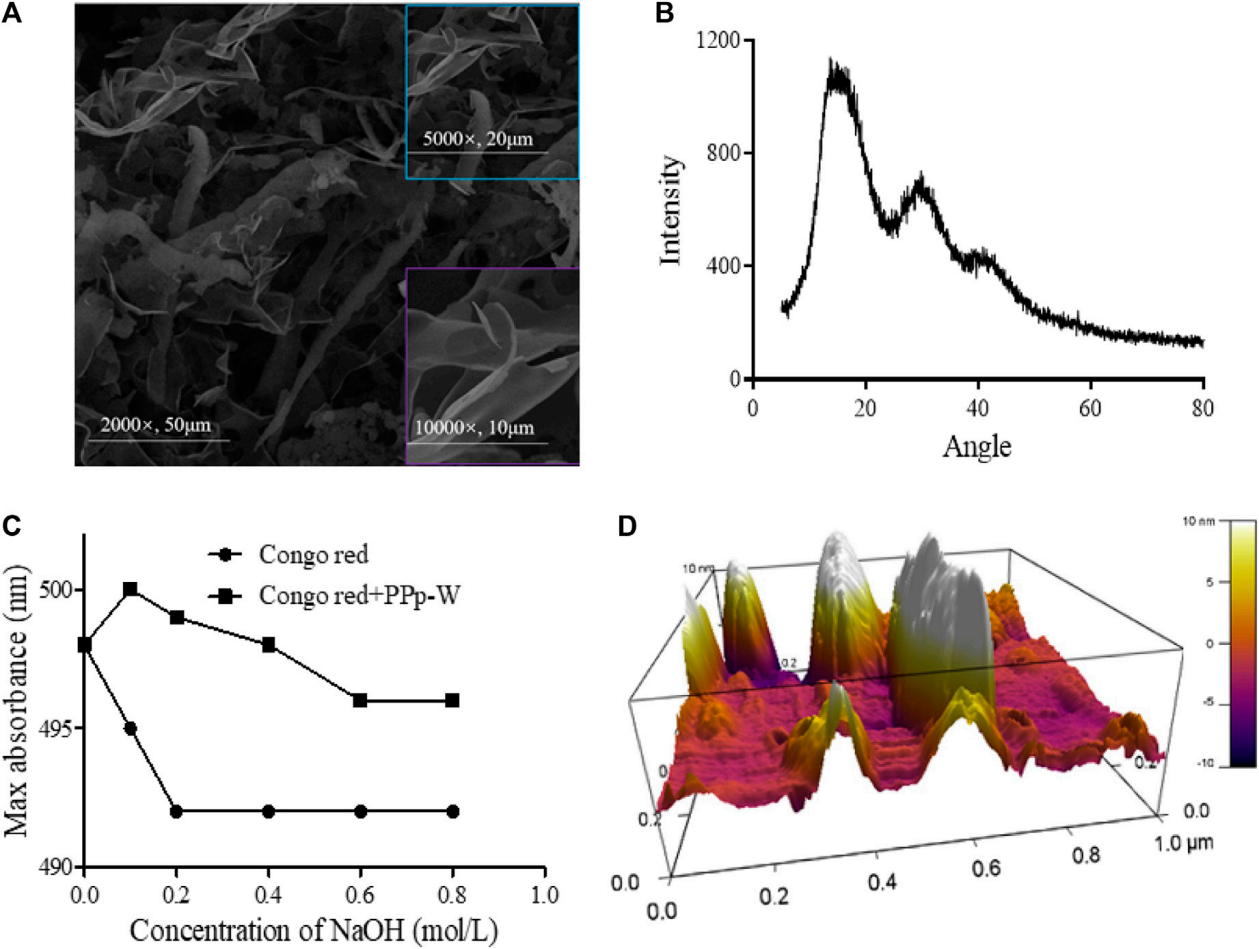
Surface morphological characterization of PPp-W from *P. placentodes*. Scanning electron microscopy image **(A)**. X-Ray diffraction spectrum image **(B)**. Congo red test–triple-helix structure analysis **(C)**. Atomic Force Microscopy image **(D)**.

In order to obtain the surface morphology of PPp-W, SEM technology was used to characterize the polysaccharide. The typical SEM image was shown in Figure 3A; PPp-W appeared to be an irregular flake shape with smooth surface, which illustrated that PPp-W was an amorphous structure. In addition, one possible explanation was that different macromolecules and ways of dehydration (such as freeze-drying and vacuum drying) affected the morphology. In addition, the surface morphology of PPp-W was similar to that of polysaccharides from *P. citrinopileatus* reported in the literature (Hao et al., 2020; Wang et al., 2021).

#### X-Ray Diffraction Spectrum Analysis

XRD had been widely used to evaluate the crystallinity of polysaccharides. The XRD spectrum of PPp-W was shown in Figure 3B. The spectrum showed a broad and low-intensity diffraction peak at approximately 15° (2θ); there were also two weaker intensity peaks at 30° and 40° (2θ), indicating that PPp-W mainly existed in amorphous form in nature. The result was consistent with the analysis result of SME, which might be related to the large and complex molecular structure of polysaccharides. According to the literatures, polysaccharides from other edible fungi have obtained similar results, for example, *Hericium erinaceus* and *Ganoderma lucidum* (Wang et al., 2018; Wu and Huang, 2021). In addition, the crystallinity of molecule was also related to its solubility, and high crystallinity would lead to the decrease of solubility in water (López-Legarda et al., 2021). The aforementioned results also indicated that PPp-W showed an amorphous structure in accordance with its high solubility in water.

#### Triple-Helix Structure Analysis

Helix-coil analysis of PPp-W at different NaOH concentration ranging from 0 to 0.8 M was shown in Figure 3C. When NaOH concentration reached 0.05 M, there was a significant redshift. With the increase of the concentration of NaOH, the λ_max_ value was decreased due to the disintegration of triple-helix structure and decrease of complex (He et al., 2021). In the same concentration of NaOH, the λ_max_ values of PPp-W–Congo red complex were significantly higher than those of the Congo red control group, which showed that PPp-W possessed a triple-helix conformation.

#### Atomic Force Microscopy Analysis

Polysaccharides usually showed irregular spherical, rod-like, and random linear chains with or without branches (Wang and Nie, 2019). The AFM image of PPp-W was shown in Figure 3D. It was clearly that PPp-W showed irregular curly structure and had branches. The heights in the range of -10–10 nm were higher than those of a single polysaccharide chain (0.1–1 nm), which also revealed that PPp-W had branches and intertwined with each other (Wang et al., 2010).

### Fine Structural Analysis of PPp-W

#### FT-IR Analysis

**FIGURE 4:**
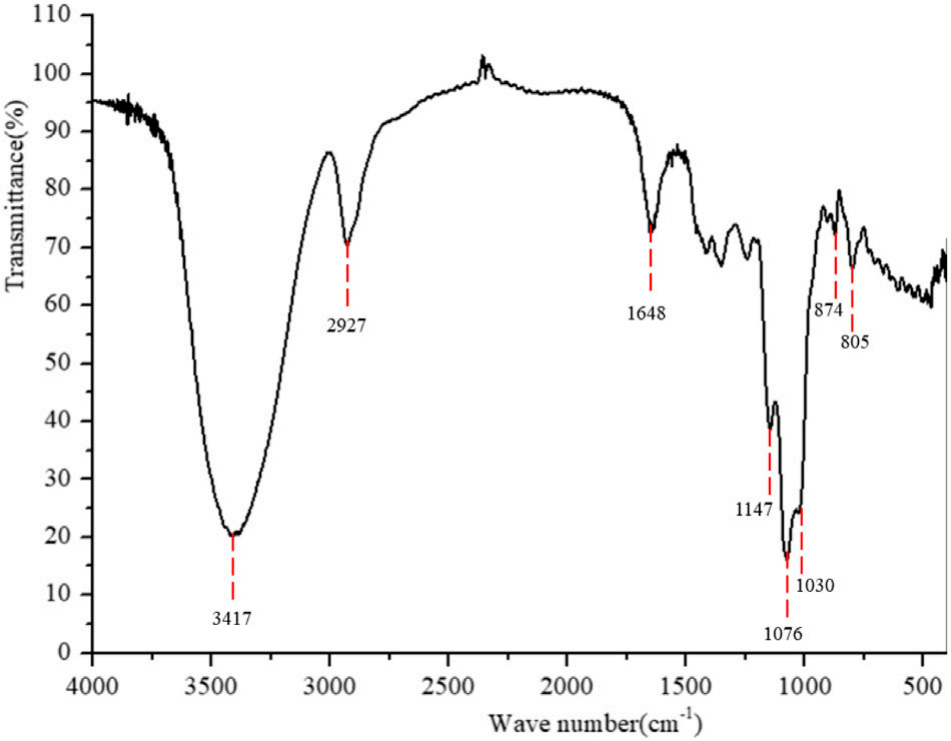
Fourier-transform infrared spectroscopy spectrum of PPp-W.

FT-IR spectrum of PPp-W was shown in Figure 4. The broad absorption peaks at 3,417 cm^−1^ responded to the stretching vibration of O-H groups; a weak and sharp absorption peak at 2,927 cm^−1^ was attributed to C–H stretching vibration, which was the characteristic absorption of polysaccharides (Wu et al., 2015). There was no uronic acid in PPp-W because of no absorption peak at 1,700 cm^−1^. The band at 1,648 cm^−1^ was due to C=O stretching vibration or the H-O-H vibration (Silverstein et al., 1992; Kaèuráková et al., 2000). The bands at 1,147, 1,076, and 1,030 cm^−1^ were attributed to the stretching vibration of pyran ring (Dong et al., 2016). In addition, the bands at 874 cm^−1^ were β-glycosidic bands, and the bands at 805 cm^−1^ were α-glycosidic bands (Azmi et al., 2012; Liu et al., 2020), indicating that PPp-W contained an α- and β-pyranose ring.

#### Glycosidic Linkage Analysis

Glycosyl linkages of PPp-W were identified by GC–MS and listed in Table 1. There were five main glycosyl linkages in PPp-W including →2,6)-Gal*p*-(1→ (23.11%), →6)-Gal*p*-(1→ (21.84%), Man*p*-(1→ (25.32%), →3)-Glc*p*-(1→ (6.81%), and Fuc*p*-(1→ (1.12%). The proportions of Gal, Man, Glc, and Fuc were basically consistent with the ratios of monosaccharide composition. On the basis of monosaccharide composition, →6)-D-Gal*p*-(1→ was derived from both 6-O-Gal*p* and 6-O-3-O-methyl-Gal*p* (NMR confirmed, see below). Therefore, we assumed that →2,6)-D-Gal*p*-(1→ and →6)-D-Gal*p*-(1→ residues formed the backbone of PPp-W, which was highly branched with D-Man*p*-(1→ as side chains linked *via* non-reducing termini. L-Fuc*p*-(1→ was positioned at the end of the sugar chain.

**TABLE 1:** Linkage patterns of PPp-W from *P. placentodes* identified by methylation and GC–MS analysis.

Methylation sugar residues	Linkage type	Relative percentage of peak area (%)	Major mass fragments (m/z)
2,4,6-Me_3_-Glc	→3)-D-Glc*p*-(1→	6.81	43, 87, 101,118, 129, 161, 190, 234, 277
3,4-Me_2_-Gal*p*	→2,6)-D-Gal*p*-(1→	23.11	43, 60, 74, 87, 100, 129, 130, 189, 190, 233
2,3,4-Me_3_-Gal*p*	→6)-D-Gal*p*-(1→	21.84	43, 71, 87, 102, 118, 129, 142, 162,173, 189, 233
2,3,4,6-Me_4_-Man*p*	D-Man*p*-(1→	25.32	43, 59, 71, 87, 102, 129, 145, 162, 205
2,3,4-Me_3_-Fuc*p*	L-Fuc*p*-(1→	1.12	43, 89, 101, 102, 115, 118, 131, 162, 175

Mass spectrum in Supplementary Figures S2A–E.

#### ^1^D/^2^D NMR Analysis

**FIGURE 5:**
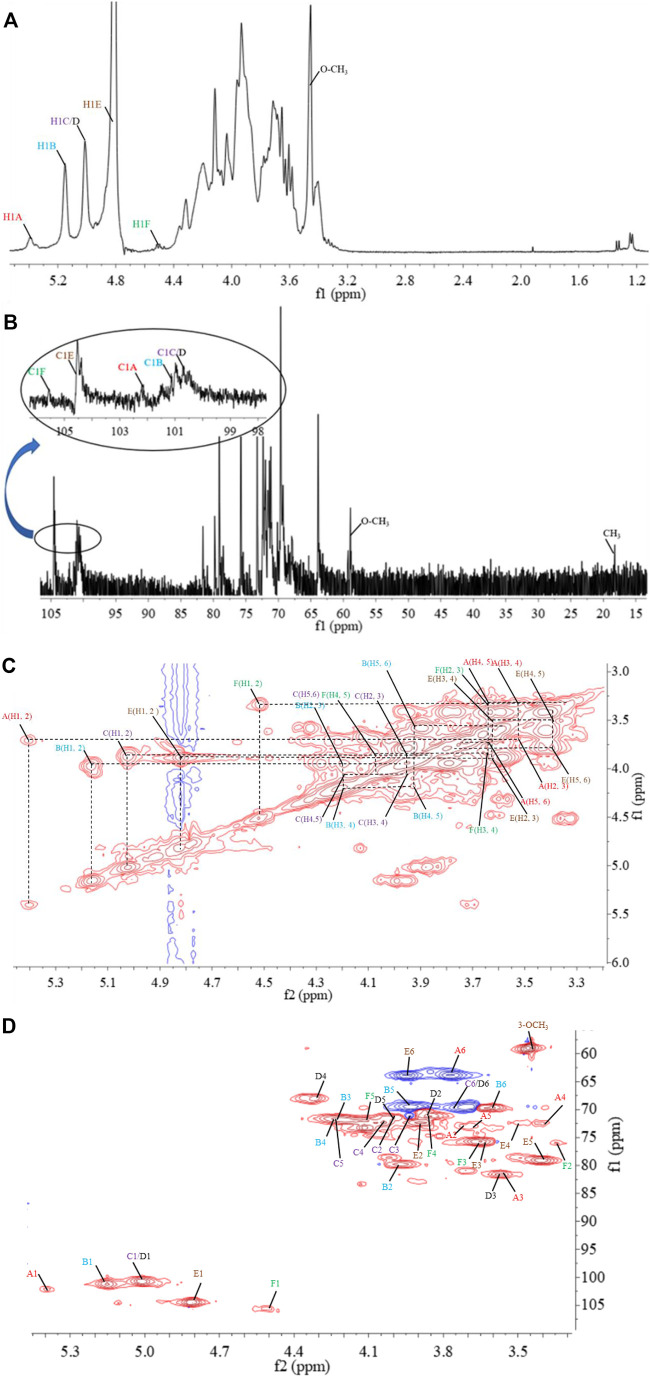
^1^D and ^2^D NMR spectrum of PPp-W. ^1^H-NMR spectrum **(A)**, ^13^C-NMR spectrum **(B)**, ^1^H–^1^H correlation spectroscopy **(C)**, ^1^H-^13^C heteronuclear single-quantum coherence spectroscopy **(D)**.

The structural feature of PPp-W was characterized using ^1^D and ^2^D NMR spectrum. The signals of anomeric protons (4.5–5.4 ppm) (Figure 5A) and anomeric carbons (90–110 ppm) (Figure 5B) suggested PPp-W contained α- and β-configurations, which was consistent with the results of FT-IR. Six signals occurred at *δ* 5.38, 5.15, 5.01, 4.94, 4.80, and 4.51 in ^1^H NMR; the signal at *δ* 4.80 overlaped with the peak of D_2_O in ^1^H-NMR. Due to the overlap, only three anomeric carbon signals were visible at *δ* 104.54, 102.47, and 100.98 in ^13^C-NMR spectrum (Figure 5B), which were resolved in the HSQC spectrum (Figure 5D). The anomeric regions showed C1/H1 signals at *δ* 5.39/102.18 (A), 5.15/101.22 (B), 5.01/100.71 (C/D), 4.80/104.49 (E), and 4.51 (4.49)/105.59 (F), and were assigned to H-1/C-1 of →3)-α-D-Glc*p*-(1→, →2,6)-α-D-Gal*p*-(1→, →6)-α-D-Gal*p*-(1→, *α*-D-Man*p*-(1→ and β-L-Fuc*p*-(1→, respectively. The identification of residues A, B, C, D, E, and F was confirmed by COSY spectrum (Figure 5C) and HSQC (Figure 5D). In addition, signals at δ 3.46 and δ 58.90 were assigned to an *O*-CH_3_ group. δ 1.21 and δ 18.26 were assigned to a CH_3_ group of Fuc. The ^1^H and ^13^C chemical shift assignments of residues A–F were summarized in Table 2.

**TABLE 2:** Assignments of ^1^H and ^13^C chemical shifts of sugar residues in PPp-W.

Residue	Chemical shifts (ppm)						
	H1/C1	H2/C2	H3/C3	H4/C4	H5/C5	H6/C6	O-Me
→3)-α-D-Glc*p*-(1→ (A)	5.38/102.32	3.7/72.7	3.57/81.59	3.37/72.39	3.65/73.21	3.82,3.71/63.86	—
→2,6)-α-D-Gal*p*-(1→ (B)	5.15/101.22	3.97/79.77	4.19/71.65	4.22/71.79	3.93/69.17	3.60,3.70/69.5	—
→6)-α-D-Gal*p*-(1→ (C)	5.01/100.71	3.87/71.16	3.95/71.44	4.03/72.29	4.20/71.85	3.68,3.92/69.44	—
→6)- 3-*O*-Me-α-D-Gal*p*-(1→ (D)	5.01/100.71	3.87/72.33	3.58/81.58	4.3/68	3.96/72.26	3.68,3.92/68.44	3.46/58.9
β*-*D*-*Man*p*-(1→ (E)	4.8/104.49	3.9/73.39	3.62/75.75	3.5/72.5	3.39/79.09	3.94,3.75/63.88	—
β*-*L*-*Fuc*p*-(1→ (F)	4.51/105.59	3.34/75.98	3.64/75.73	3.82/71.15	4.13/72.06	1.23/18.26	—

The weaker anomeric protons of residue A at *δ* 5.38/102.32 showed an α-linked fragment and was assigned to →3)-α-D-Glc*p*-(1→ based on the data of methylation and relevant literature (Li et al., 2016). The H-2∼H-6 were observed from COSY spectrum (Figure 5C), and the corresponding carbons were visible and assigned from HSQC spectrum (Figure 5D). The chemical shifts of H-2/C-2∼H-6(6′)/C-6 of residue A were found at *δ* 3.7/69.63, 3.53/81.60, 3.37/72.39, 3.65/73.21, and 3.80 (3.71)/63.86, respectively.

For residue B, the chemical shift of H-2 was observed at δ 3.97 from H-1 in the COSY spectrum, and C-2 was assigned to be δ 79.79 achieved by the HSQC spectrum. Other resonances of H-3, H-4, H-5, H-6, and H-6′ were found to be δ 4.19, 4.22, 3.93, 3.60, and 3.70 according to the COSY and HSQC spectruma. The ^13^C chemical shifts of C-3, C-4, C-5, and C-6 were clearly observed at δ 71.65, 71.79, 69.17, and 69.5, respectively. Compared with literature (Maity et al., 2020), residue B was identified as →2,6)-α-D-Gal*p*-(1→.

Based on the anomeric signals at *δ*5.01/100.71, the residues C and D were *α*-linked fragments. For residue C, the chemical shift of H-2 was observed by the correlations of H-1/H-2 (*δ* 5.01/3.87). Other signals were obtained by the cross-peaks of H-2/H-3 (δ 3.87/3.95), H-3/H-4 (δ 3.95/4.03), H-4/H-5 (δ4.03/4.20), and H-5/H-6 (6′) [δ 4.20/3.92 (3.68)]. Based on HSQC correlations, the corresponding carbon signals were clearly observed at δ 71.16, 71.44, 72.29, 71.85, and 69.88, respectively. The downfield shift of C-6 signal suggested the glycosylation of residue C at *O*-6 confirmed by HMBC correlation H-1/C-6 at δ 5.01/69.88. Residue C was signed as galacto configuration (Gheysen et al., 2008) but for residue D, most of the protons and carbons had similar distribution with residue C. Interestingly, the chemical shift of *O*-CH_3_ (δ 3.46/58.90) was assigned from the HSQC spectrum, and ^1^H resonances for *O*-CH_3_ correlated with C-3 (δ 3.46/81.59) of residue D in the HMBC spectrum showed that *O*-CH_3_ was located on residue D, and C-3 was assigned to H-4/C-3 (δ 4.3/81.59) in HMBC spectrum (Figure 5A). Therefore, residue C was ascribed to →6)-α-D-Gal*p*-(1→, and residue D was assigned to →6)-3-*O*-Me-*α*-D-Gal*p*-(1→ (Zhang et al., 2013; Oliveira et al., 2019). The aforementioned results confirmed the existence of 3-O-Me-galactose and further confirmed the results of monosaccharide composition and methylation analysis.

For residue E, the anomeric signals were found at δ 4.80/104.49, which showed the presence of β-link in residue E. The signals from H-1/C-1 to H-6/C-6 were identified from COSY and HSQC spectrum (Table 2). The β-configuration of residue E was also inferred by the H-5 and C-5 chemical shifts at δ 3.39 and 79.09 ppm (compared published data δ 3.82/73.34 or δ 3.38/77.00 for β-mannopyranose), respectively (Jansson et al., 1989; Oliveira et al., 2019; Ellefsen et al., 2021).

A peak at δ 18.26 ppm belonged to C-6 of fucose in the ^13^C-NMR spectrum, while the strong peak observed at around δ 1.21 ppm in the ^1^H NMR spectrum was a typical signal of H-6 of fucose. The chemical shifts of H-2, H-3, H-4, and H-5 of residue D were δ 3.34, 3.64, 3.82, and 4.13 ppm, according to the COSY spectrum (Figure 5C). The corresponding ^13^C NMR signals were assigned to be δ 75.98, 75.73, 71.15, and 72.06 from HSQC spectrum (Figure 5D). According to the relevant literature (Xiao et al., 2021), residue F was considered as β-L-Fuc*p*-(1→.

**FIGURE 6:**
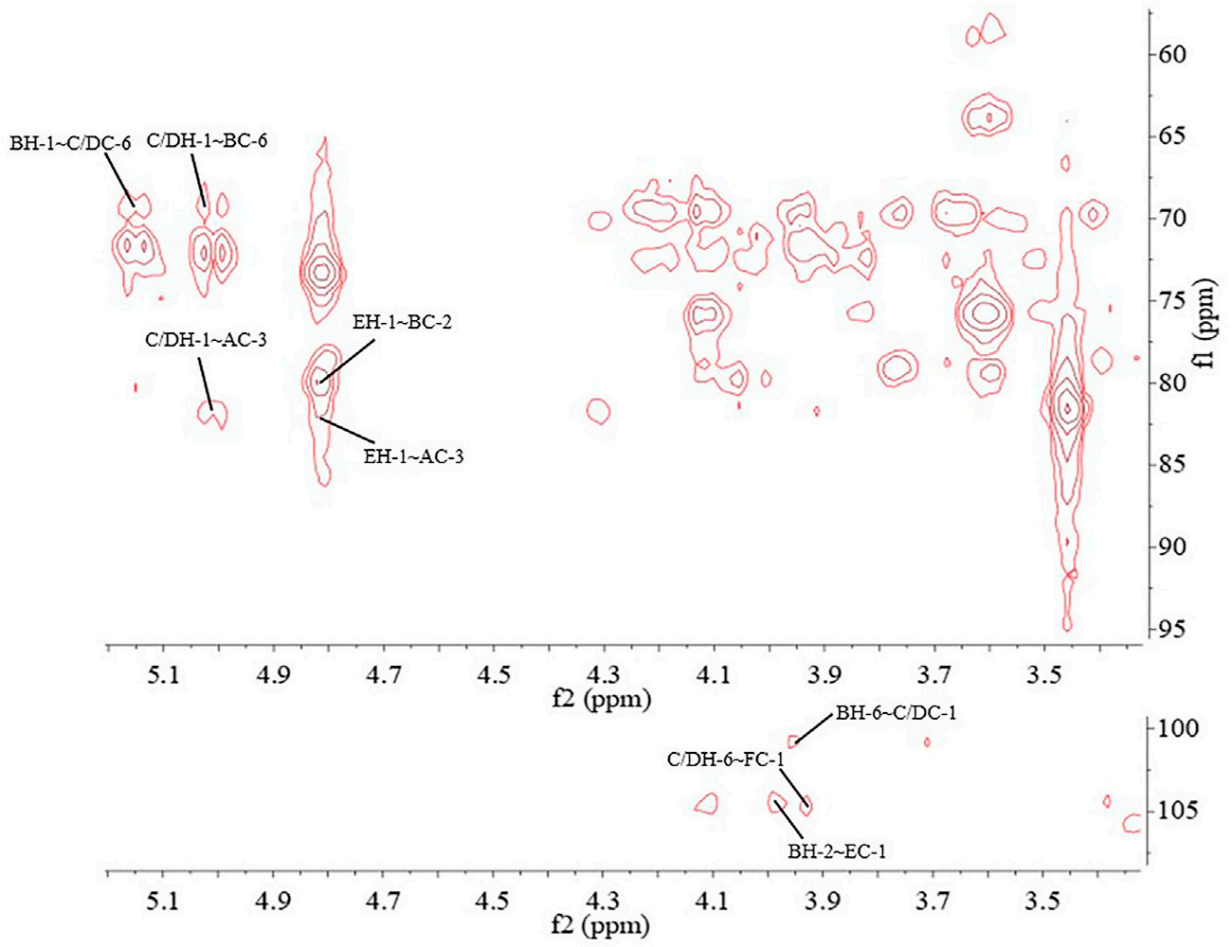
^1^H-^13^C heteronuclear multiple-bond spectroscopy (HMBC) spectrum of PPp-W.

**FIGURE 7:**
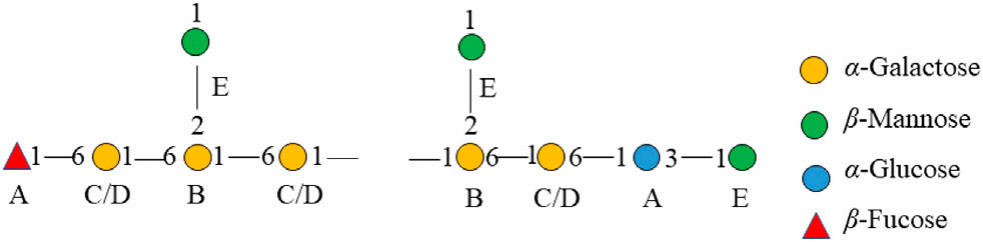
Fragment of the chemical structure of PPp-W.

The major proton/carbon connectivities among residues A∼F were revealed by ^1^H-^13^C HMBC spectrum (Figure 6; Table 3). The correlations between H-1 of residue C/D and C-6 of residue B, H-1 of residue B and C-6 of residue C/D, and H-1 of residue E and C-2 of residue B indicated that *O*-1 of → 6)-α-D-Gal*p*-(1→ was interconnected to *O*-6 of → 2, 6)-α-D-Gal*p*-(1→, O-1 of → 2, 6)-α-D-Gal*p*-(1→ was interconnected to O-6 of → 6)-α-D-Gal*p*-(1→, and O-1 of β-D-Man*p*-(1→ was interconnected to O-2 of → 2, 6)-α-D-Gal*p*-(1→. Based on the proportion of monosaccharide fragments, we could infer that →1, 6)-linked-Gal*p* and →1, 2, 6)-linked-Gal*p* were the main chains, and β-D-Man*p*-(1→ was the branch chain. In addition, the correlations between H-1 of residue C/D and C-3 of residue A, and H-6 of residue C/D and C-1 of residue A showed that O-1 of →1, 6)-linked-Gal*p* was linked to O-3 of →1, 6)-linked-Glc*p*, and O-1 of →1, 6)-linked-Glc*p* was linked to O-6 of →1, 6)-linked-Gal*p*. The correlations between H-1 of residue E and C-3 of residue A, and H-6 of residue C/D and C-1 of residue F showed that O-1 of β-D-Man*p*-(1→ was linked to O-3 of →1, 6)-linked-Glc*p*, and O-6 of →1, 6)-linked-Gal*p* was linked to O-1 of β-L-Fuc*p*-(1→. Therefore, it could be inferred that there was a fragment containing 1,3-linked-Glc*p* in the main chain of PPp-W. The links of carbon chain were shown in Figure 7.

**TABLE 3:** Two-and three-bond ^1^H–^13^C correlations for the PPp-W in the HMBC spectrum.

Residue	Proton	Proton correlation^a^
→3)-α-D-Glc*p*-(1→(A)	H-1	72.7 (**A**: C-2)
	H-1	69.5(**B**: C-6); **69.44(C/D: C-6)**
→2,6)-α-D-Gal*p*-(1→(B)	H-2	71.65(**B**: C-3); 101.22(**B**: C-1); **104.49(E: C-1)**
—	H-6	**100.71 (C/D: C-1)**
→6)-α-D-Gal*p*-(1→(C)/→6)-3-O-Me-α-D-Gal*p*-(1→(D)	H-1	69.44(**C**: C-6); 71.16(**C**: C-2); **69.5 (B: C-6); 81.6 (A: C-3)**
—	H-6	71.85(**C**: C-5); **105.59 (F: C-1)**
β-D-Man*p*-(1→ (E)	H-1	73.29(**E**: C-2); **79.77 (B: C-2); 81.6(A: C-3)**

a Inter-residue correlations are shown in bold font.

Overall, PPp-W was a 3-*O*-methylated heteroglycan and showed an irregular curly sheet with triple-helix conformation. It was reported that 3-*O*-Me-D-Gal*p* was also found in *P. eryngii*, *P. geesteranus*, and *P. pulmonarius* (Smiderle et al., 2008; Zhang et al., 2013a; Zhang et al., 2013b; He et al., 2016; Brito et al., 2018; Yan et al., 2019). The summary of 3-O-Me -D-Gal *p* polysaccharides from *Pleurotus* spp. were showed in Table 4. Interestingly, PPp-W had the same molecular weight as polysaccharide PCP60W from *P. citrinopileatus*, and PCP60W was shown to be a linear partially 3-*O*-methylated-α-galactopyranan comprised of 6-linked galactose, 6-linked 3-*O*-methyl galactose, and 4-linked glucose in a ratio of 3.0:1.0:0.6, but they had different monosaccharide compositions (He et al., 2016). It was found that PPp-W also contained 3-*O*-Me-Gal fragment, but the link fragments were still very different, which might be related to the source and processing method.

**TABLE 4:** A summary of 3-*O*-Me-*D*-Gal*p* polysaccharides from *Pleurotus* spp.

Source	Extraction method	Mw (KDa)	Monosaccharide composition	Structural characteristics	References
*P. eryngii* fruiting bodies	Extracted with distilled water three times at 100°C for 4 h each time, purified by DEAE-cellulose and Sepharose CL-6B columns	21.4	Gal (43.8%), Man (39.3%), methyl-Gal (11.7%), and Glc (9.2%)	Main chain: →6)-α-D-Gal*p*-(1→ and 3-O-Me-D-Gal*p*, branched at O-2 with single t-β-D-Man*p* as the major side chain	Yan et al. (2019)
*P. citrinopileatus* fruiting bodies	Extracted three times (2 h each time) with boiling water, purified by diethylaminoethyl sepharose fast flow and Sephacryl S-300 gel columns	27.4	Glc, Gal, and an unknown sugar	→6)-α-D-Gal*p*-(1→, →6)-α-3-O-Me-D-Gal*p*-(1→, and →4)-α-D-Glc*p*-(1→	He et al. (2016)
*P. geesteranus* fruiting bodies	Extracted with distilled water for 2 h at 100°C, purified by DEAE-sepharose fast flow and high-resolution fephacryl S-300	13	Man, Glc, and Gal, along with an amount of 3-O methylgalactose	Main chain: →6)-α-D-Gal*p*-(1→ and α-D-3-O-Me-D-galactosyl unit backbone with α-D-mannosyl unit on O-2 of the 2,6-di-O-substituted-D-galactosyl units	Zhang et al. (2013a)
*P. eryngii* fruiting bodies	Extracted with boiling distilled water thrice (2 h for each), purified by DEAE Sepharose Fast Flow colum, high-resolution sephacryl S-300 and S-100 gel-permeation chromatograph	18.8	Gal, Man	→6)-α-D-Gal*p*-(1→ backbone with a β-D-mannosyl unit on O-2 of the 2,6-di-O-substituted-D-galactosyl units, α-(1→6)-3-O-Me-D-galactopyranan backbone with a terminal α-D-3-O-Me-D-galactosyl unit	Zhang et al. (2013b)
*P. pulmonarius*	Extracted with water at 25°C for 6 h, treatment with Fehling solution and ultrafiltration	23.9	Fuc (2%), Xyl (1%), Man (27%), 3-O-methyl-galactose (15%), Gal (47%), and Glc (8%)	Main chain: →6)-α-D-Gal*p*-(1→ and 3-O-methyl-α-D-galactopyranosyl, partially substituted at O-2 by β-D-mannopyranosyl non-reducing ends	Smiderle et al. (2008)
*P. citrinopileatus* fruiting bodies	Extracted with water at 10°C for 6 h, treatment with Fehling solution and deionized with ion-exchange resins	37.6	3-O-Me-Gal and Gal (1: 2 M ratio)	Linear (1/6)-linked α-galactopyranans partially 3-O-methylated	Brito et al. (2018)
		28.5	3-O-Me-Gal and Gal (1:1 M ratio)		

#### Anticoagulant Activity of PPp-W

**FIGURE 8:**
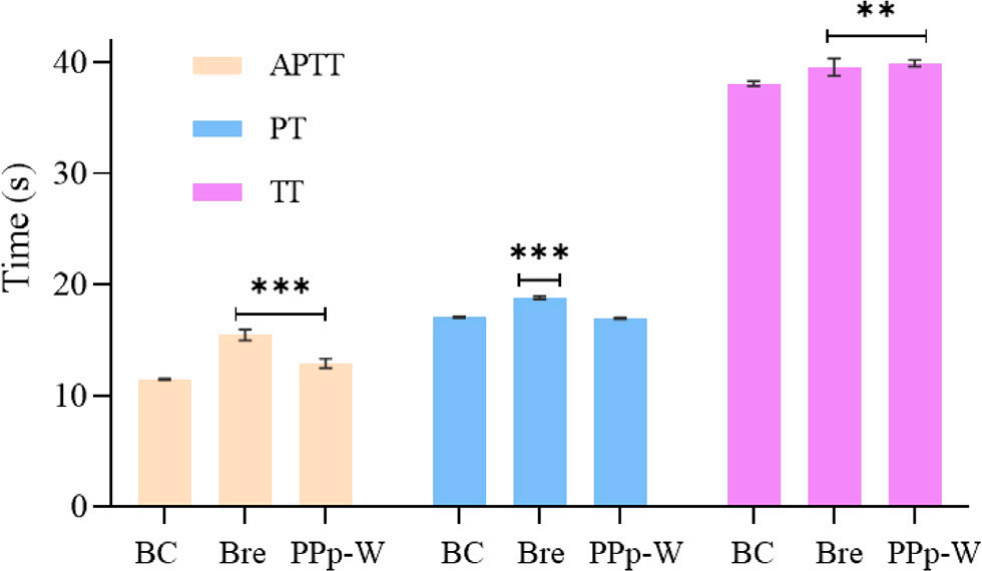
Effects of PPp-W on activated partial thromboplastin time (APTT), prothrombin time (PT), and thrombin time (TT) *in vitro* (*n* = 3) (Compared with BC, ***p < 0.001 **p < 0.01 *p < 0.05).

The effects of PPp-W on APTT, PT, and TT were shown in Figure 8. Compared with the BC, breviscapine as an anticoagulant could significantly prolong APTT, PT, and TT (*p* < 0.01 or *p* < 0.001), and PPp-W could significantly prolong APTT and TT (*p* < 0.01 or *p* < 0.001), which proved that PPp-W had anticoagulant activity. In clinic, APTT, PT, and TT were often used as indexes to evaluate the coagulation function of the body. As an endogenous coagulation pathway, APTT was related to coagulation factors VIII, IX, XI, and prekallikrein, while PT, an exogenous coagulation pathway, was related to coagulation factors I, V, VII, and X. TT mainly reflected the degree of conversion of fibrinogen to fibrin (Xie et al., 2017; Yin et al., 2021). It could be seen that the anticoagulant activity of PPp-W was related to endogenous coagulation pathway and thrombin-mediated fibrin formation.

Previous studies have shown that there were many factors affecting the biological activity of polysaccharides including structure, configuration, molecular weight, branching structure, and the position of sulfate group (Yang et al., 2002; Yang et al., 2005; Raposo and Morais, 2015). Based on the structure analysis of PPp-W, PPp-W was mainly composed of Man and Gal, and had a triple-helix conformation; the molecular weight was 27.4 kDa. The anticoagulation activity of PPp-W might be related to its monosaccharide composition, molecular weight, and triple-helix structure, and the structure–activity relationship needed to be further studied.

## Conclusion

In this study, 3-*O*-methylated heteroglycan (PPp-W) was identified from fruiting bodies of *P. placentodes*. PPp-W showed irregular curly sheets with triple-helix conformation, and had a main chain composed of → 6)-α-D-Gal*p*-(1→, →6)*-*α-3-*O*-Me-D-Gal*p*-(1→ and →2, 6)-α-D-Gal*p*-(1→, partially substituted at *O*-2 and *O*-6 by non-reducing ends of β*-*D-Man*p*-(1→ and β*-*L-Fuc*p*-(1→. In addition, there were small amounts of 1,3-linked-Glc*p* in the backbone. PPp-W could significantly prolong APTT and TT to show anticoagulant activity. This study made up for the blank in the study of the structure of polysaccharides from *P. placentodes*. Based on the structure, we will further study the activity and mechanism of polysaccharides from *P. placentodes*, which may be suitable for functional foods and natural medicine as an anticoagulant ingredient.

## Data Availability

The original contributions presented in the study are included in the article/Supplementary Material; further inquiries can be directed to the corresponding authors.
