# Supplementary figures and images for: Structural Characterization and Anticoagulant Activity of a 3-O-Methylated Heteroglycan From Fruiting Bodies of Pleurotus placentodes
Source: Front Chem. 2022 Jan 27;10:825127. doi: 10.3389/fchem.2022.825127 (PMC8829048; doi:10.3389/fchem.2022.825127)

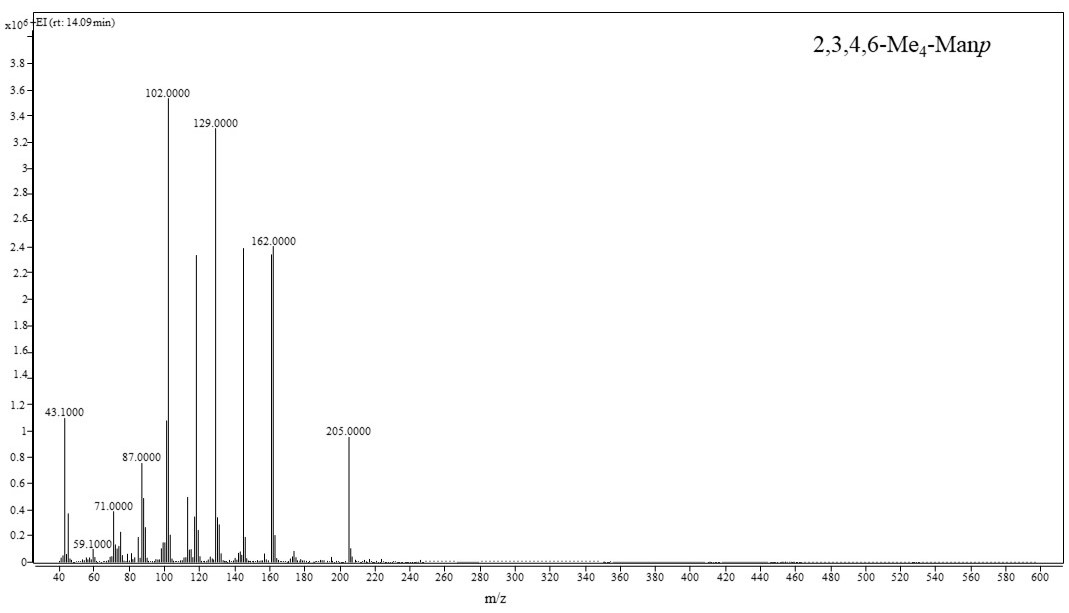

Supplement: Supplementary file 1 [file Image3.JPEG]

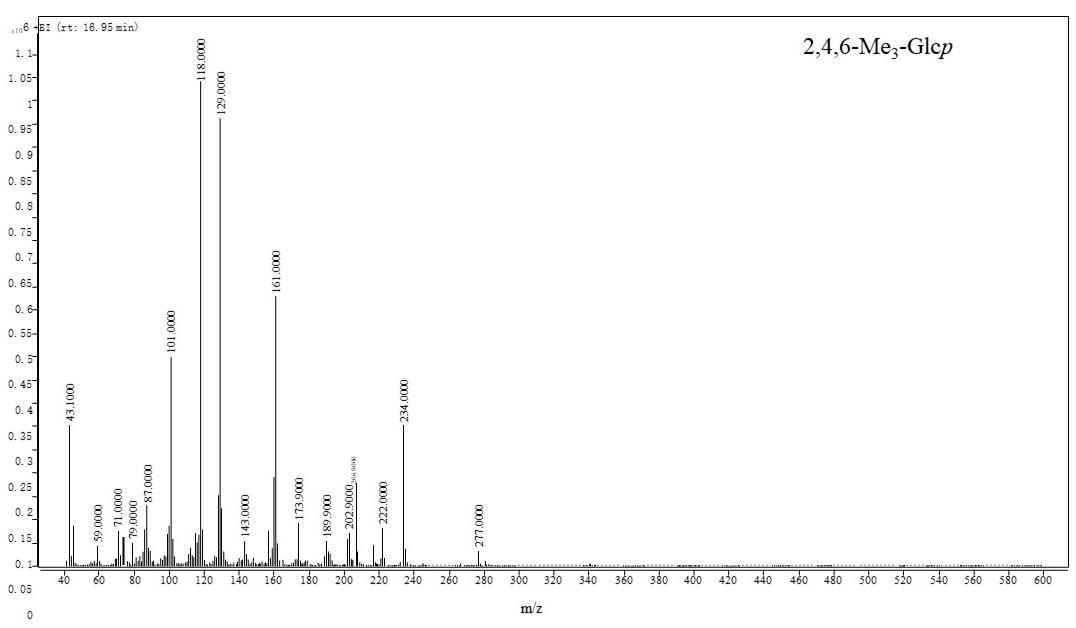

Supplement: Supplementary file 2 [file Image4.JPEG]

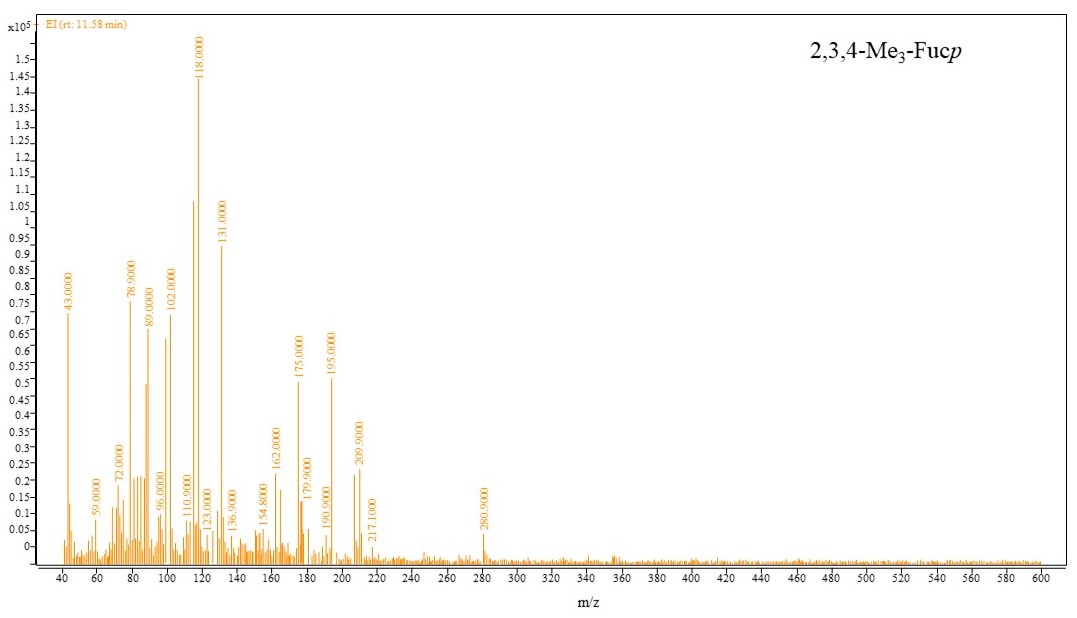

Supplement: Supplementary file 3 [file Image2.JPEG]

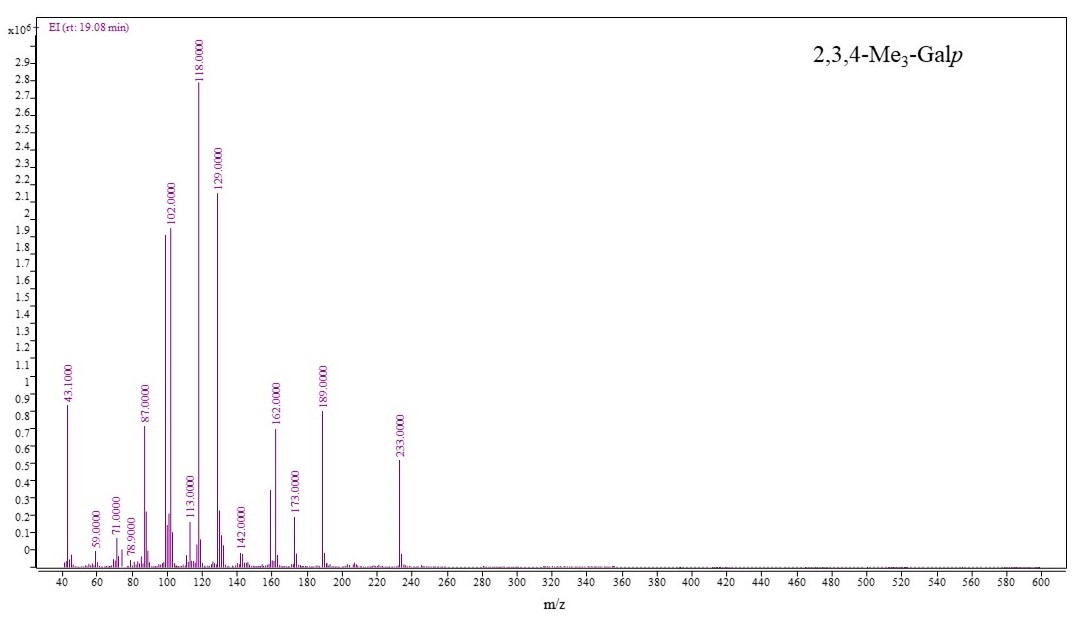

Supplement: Supplementary file 4 [file Image5.JPEG]

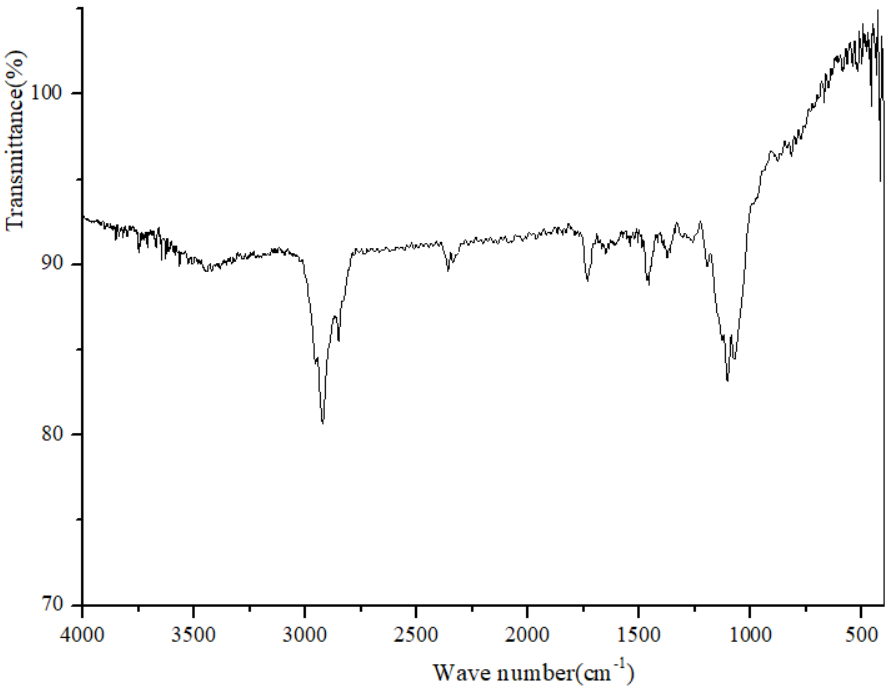

Supplement: Supplementary file 5 [file Image1.PNG]

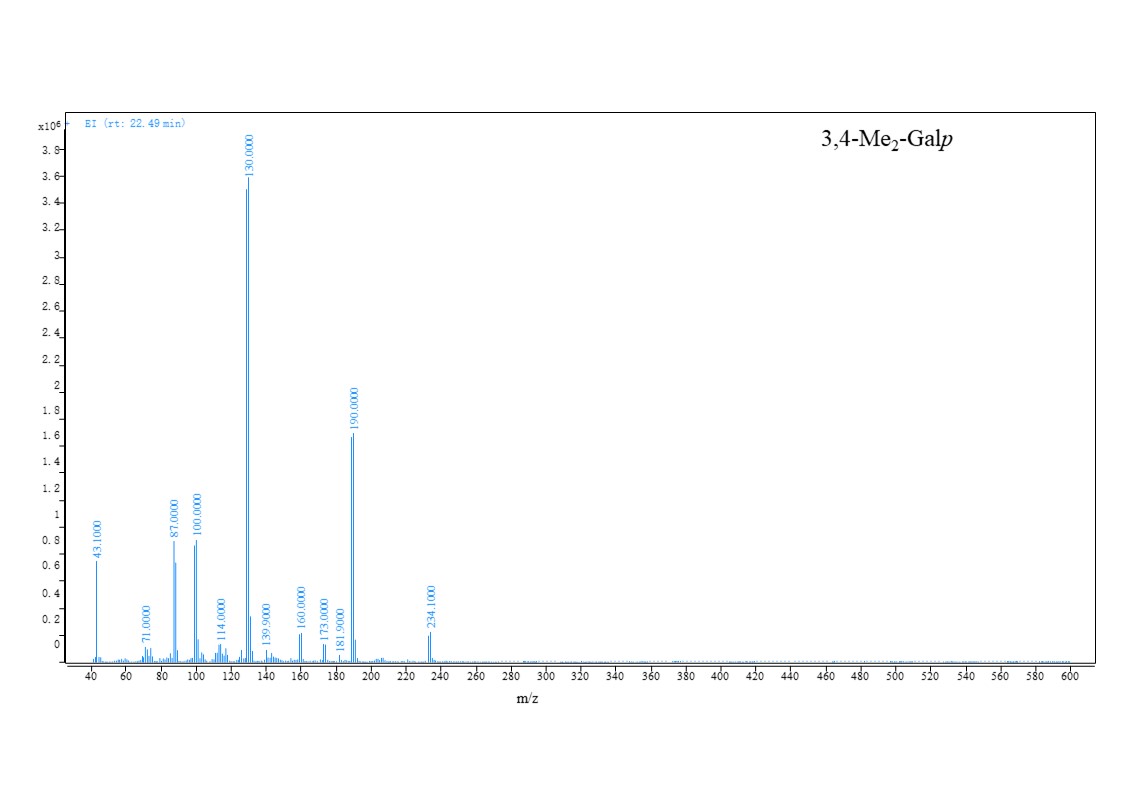

Supplement: Supplementary file 6 [file Image6.JPEG]
